# Vocabulary Size Is a Key Factor in Predicting Second Language Lexical Encoding Accuracy

**DOI:** 10.3389/fpsyg.2021.688356

**Published:** 2021-07-22

**Authors:** Danielle Daidone, Isabelle Darcy

**Affiliations:** ^1^Department of World Languages and Cultures, University of North Carolina Wilmington, Wilmington, NC, United States; ^2^Department of Second Language Studies, Indiana University, Bloomington, IN, United States

**Keywords:** lexical encoding, vocabulary size, phonological short-term memory, inhibitory control, attention control, L2 perception, L2 Spanish

## Abstract

This study investigates the relationship between the accuracy of second language lexical representations and perception, phonological short-term memory, inhibitory control, attention control, and second language vocabulary size. English-speaking learners of Spanish were tested on their lexical encoding of the Spanish /ɾ-r/, /ɾ-d/, /r-d/, and /f-p/ contrasts through a lexical decision task. Perception ability was measured with an oddity task, phonological short-term memory with a serial non-word recognition task, attention control with a flanker task, inhibitory control with a retrieval-induced inhibition task, and vocabulary size with the X_Lex vocabulary test. Results revealed that differences in perception performance, inhibitory control, and attention control were not related to differences in lexical encoding accuracy. Phonological short-term memory was a significant factor, but only for the /r-ɾ/ contrast. This suggests that when representations contain sounds that are differentiated along a dimension not used in the native language, learners with higher phonological short-term memory have an advantage because they are better able to hold the relevant phonetic details in memory long enough to be transferred to long-term representations. Second language vocabulary size predicted lexical encoding across three of the four contrasts, such that a larger vocabulary predicted greater accuracy. This is likely because the acquisition of more phonologically similar words forces learners’ phonological systems to create more detailed representations in order for such words to be differentiated. Overall, this study suggests that vocabulary size in the second language is the most important factor in the accuracy of lexical representations.

## Introduction

Models of second language (L2) speech perception have typically focused on the effect of the first language (L1) at the level of phonetic or phonological categories (e.g., Flege, 1995; Best and Tyler, 2007), with the implicit assumption in the field being that the accuracy of category perception directly translates to the accuracy of these sounds in the lexicon, that is, of lexical representations. However, recent empirical studies have found variation in the relationship between accuracy in perception and lexical representations (Elvin, 2016; Simonchyk and Darcy, 2017; Llompart, 2021b), while others have found that even accurate perception is not a guarantee of accurate lexical encoding (e.g., Darcy et al., 2013; Daidone and Darcy, 2014; Amengual, 2016). Thus, the relationship between perception ability and lexical encoding is not straightforward. This suggests that the phonological forms of words in the L2 mental lexicon (i.e., L2 phonolexical forms) may generally be less detailed, or “fuzzy” (Hayes-Harb and Masuda, 2008; Cook, 2012; Darcy et al., 2013), above and beyond what would be expected from perception ability alone. Consequently, there must be other factors at play that influence learners’ ability to encode the sounds of L2 words in long term memory. Identifying these factors is important theoretically, as examining such individual differences can give a window into the mechanisms necessary for the establishment of lexical representations. Additionally, identifying these factors is the first step in determining how to aid learners in acquiring more accurate L2 lexical representations.

It is likely that variability in lexical encoding accuracy may be due to learners’ differing abilities to select the relevant information in the signal, hold sounds in memory, or reduce the influence of their L1 phonological grammar during word learning. Previous studies have shown that phonological short-term memory (e.g., Aliaga-García et al., 2011), inhibitory control (e.g., Lev-Ari and Peperkamp, 2013, 2014; Darcy et al., 2016), attention control (e.g., Darcy et al., 2014), and L2 vocabulary size (e.g., Bundgaard-Nielsen et al., 2011, 2012) are all possibly involved in enhancing the processing of L2 sounds or modulating cross-linguistic phonological influence on perception or production. However, the link between learners’ phonological short-term memory, inhibitory control, attention control, and L2 vocabulary size and the accuracy of their lexical representations has largely been unexplored.

### Background

For native speakers, the representations of words accurately reflect the sound system of the language being processed, and the process of selecting appropriate representations in the lexicon is efficient and largely error-free. For L2 learners, however, this is not necessarily the case, as they may have difficulty in both the accurate storage and processing of L2 words. While this is often attributable to difficulty with the perception of novel L2 contrasts, the relationship between accuracy in perception and accuracy in lexical encoding has been shown to vary by proficiency level and the language pairing or contrasts under investigation (Elvin, 2016; Simonchyk and Darcy, 2017; Llompart, 2021b). For example, Simonchyk and Darcy (2017) examined the relationship between perception and lexical encoding for plain versus palatalized consonants for English-speaking learners of Russian at different levels of proficiency. They found that there was no relationship between intermediate learners’ error rates in an ABX perception task and their error rates in an auditory word-picture matching task. In contrast, for advanced learners, higher ABX error rates were positively correlated with higher errors rates in the auditory word-picture matching task. In other words, those learners with better perception were also more accurate at lexical encoding, but only if they were at an advanced proficiency level. Llompart (2021b) reported the opposite result, finding that differences in perception ability were only significant predictors of lexical encoding of words with /ε/ and /æ/ for his intermediate German-speaking learners of English, not his advanced learners. Thus, a learner’s perception ability alone is not sufficient to predict the accuracy of their lexical representations.

Additionally, even learners with accurate perception experience difficulties with L2 lexical encoding (Sebastián-Gallés and Baus, 2005; Díaz et al., 2012; Darcy et al., 2013; Amengual, 2016). In Darcy et al. (2013), ABX tasks determined that English-speaking learners of German were able to discriminate front and back rounded vowels, and English-speaking learners of Japanese were able to discriminate singleton and geminate consonants. Nevertheless, in a lexical decision task, intermediate learners in both groups and advanced Japanese learners had trouble rejecting non-words if the real word contained a new L2 category; for example, they accepted ^∗^*kipu* /kipɯ/ as a word when the real word is *kippu* /kippɯ/ ‘ticket’. Even highly proficient early bilinguals have been found to exhibit this tendency to perform less well on lexical tasks than would be expected from their accuracy on perceptual tasks. Amengual (2016) reported that Spanish-Catalan bilinguals had high accuracy on forced-choice identification and AX discrimination tasks, but had difficulty rejecting non-words with the incorrect vowel from the /e-ε/ contrast. Another study on Spanish-Catalan bilinguals by Sebastián-Gallés and Baus (2005) had participants complete a categorical perception task, which looked at their perceptual boundary for /e-ε/; a gating task, which examined how much of the word was necessary to be heard for it to be correctly chosen; and a lexical decision task, which looked at whether participants could correctly reject non-words with /e/ and /ε/ switched. They found that while 68.3% of the participants scored within the native Catalan range for the perception task, only 46.6% did so for the gating task. A mere 18.3% had native-like performance on the lexical decision task. These results show that exhibiting a native-like perceptual boundary between these two vowels in isolation did not entail that they were represented correctly in words. Díaz et al. (2012) found similar results when testing Dutch-speaking late learners of English. While almost half the L1 Dutch participants in their study scored within the native range for a categorization task testing the English /æ-ε/ contrast, only a few scored within the native range for tasks tapping lexical knowledge, suggesting that for most participants their lexical representations containing /æ/ or /ε/ were not as accurate as their perception of those vowels.

While it is always possible that the discrimination tasks researchers have used were not sensitive enough to expose learners’ continued difficulties with novel L2 sounds, even L2 words that do not contain confusable phonemes have been shown to be less effectively recognized (Cook, 2012; Cook and Gor, 2015; Cook et al., 2016). Cook et al. (2016) administered a translation judgment task to English-speaking learners of Russian in which participants heard a word such as /malatok/ ‘hammer’ followed by the English translation (< HAMMER >) presented visually. Participants then decided whether the English word was the correct translation of the Russian word. In some cases, the auditory stimulus was not the translation of the following English word, but rather a phonologically similar word, such as /malako/ ‘milk.’ Importantly, these words did not differ based on contrasts that were difficult for L2 learners. They found that unlike native speakers, learners were willing to accept phonologically similar words as a match to the translation, and the more similar the words were to the correct translation, the more likely they were to accept them.

It is clear that the L1 phonological system affects learners’ ability to accurately encode L2 words. However, given that even accurate perception does not always lead to accurate lexical encoding, the nature of L2 phonolexical representations cannot be explained solely by interference from the L1 phonological system. Therefore, other factors must also be playing a role in the accuracy of these representations. We investigate the following four factors in addition to perception: phonological short-term memory, inhibitory control, attention control, and vocabulary size in the second language. Reasons that make these factors good candidates to impact the accuracy of L2 lexical representations are outlined below.

#### Phonological Short-Term Memory

One cognitive ability that may be related to learners’ individual differences in L2 phonolexical representations is phonological short-term memory (PSTM), which is the phonological loop component of working memory. The phonological loop allows for the storage and manipulate of auditory information; it is capable of maintaining auditory memory traces for up to a few seconds before they decay, unless they are renewed by sub-vocal articulatory rehearsal (Baddeley, 2003).

Researchers that have examined the relationship between individual differences in PSTM and perception have reported that learners with higher PSTM generally have more accurate perception of vowels and consonants (MacKay et al., 2001; Aliaga-García et al., 2011; Lengeris and Nicolaidis, 2014; Cerviño-Povedano and Mora, 2015; Darcy et al., 2015). For example, Lengeris and Nicolaidis (2014) found that Greek learners of English with higher PSTM were more accurate at identifying English consonants, in noise and in quiet. Aliaga-García et al. (2011) reported that bilingual Catalan-Spanish learners in the high PSTM group had more accurate perception of synthesized English vowel stimuli than did the learners in the low PSTM group, although Safronova and Mora (2012) did not reproduce this finding. The results of these studies suggest that higher PSTM may help learners develop more target-like cue-weighting and therefore more native-like perception, as suggested by Cerviño-Povedano and Mora (2015). They reported that Spanish-speaking learners of English with higher PSTM were less likely to over-rely on duration as a cue to the English /i-ɪ/ contrast.

PSTM has also been shown to be related to accuracy and gains over time in L2 production (O’Brien et al., 2007; Nagle, 2013; Mora and Darcy, 2016). For example, Nagle (2013) examined the relationship between the pronunciation ratings given to English-speaking learners of Spanish and their PSTM. He found a moderate positive correlation between the two, such that higher PSTM was related to higher pronunciation ratings from native Spanish speakers. A positive relationship between PSTM and pronunciation accuracy was also evidenced by Mora and Darcy (2016) for Spanish-speaking learners of English.

Overall, the majority of studies have shown that higher PSTM is related to more accurate L2 perception and production, accounting for a small but significant portion of the variance or evidencing at least a moderate correlation. Researchers hypothesize that this is because learners who have a greater ability to encode and maintain detailed and accurate short-term representations of sounds subsequently transfer these more target-like representations to long-term memory and into lexical representations. In turn, the enhanced development of new L2 phonetic categories stems from these more accurate long-term representations of words (Speciale et al., 2004; Nagle, 2013). While this proposed connection between PSTM and lexical encoding has not previously been examined empirically, this hypothesis suggests that there should be a positive relationship between variance in PSTM and the accuracy of L2 phonolexical encoding in the current study.

#### Inhibitory Control

Another factor that may affect lexical encoding is inhibitory control. In general, inhibitory control is a type of executive function that allows an individual to suppress a dominant internal response or override the pull of an external stimulus and instead respond in a more appropriate manner (Diamond, 2013). Various taxonomies of inhibition, interference control, or executive functions more broadly have been proposed, with a lack of general agreement between studies on the use of terms (Nigg, 2000; Friedman and Miyake, 2004; Miyake and Friedman, 2012). For the present study, the most relevant type of inhibition is that referred to by Friedman and Miyake (2004) as *Resistance to Distractor Interference*, or “the ability to resist or resolve interference from information in the external environment that is irrelevant to the task at hand” (p. 104). Although not necessarily termed as such within the studies themselves, a body of work has found that the results of tasks testing resistance to distractor interference is related to the amount of interference between bilinguals’ L1 and L2 phonology in production and perception.

Using a retrieval-induced inhibition task, Lev-Ari and Peperkamp (2013) investigated the relationship between inhibitory control and L2 influence on the L1 phonology. They found that English-French bilinguals with lower inhibitory skill produced the voiceless stops /p t k/ with shorter, more French-like VOT values when speaking English. Those with lower inhibitory skill also categorized more tokens along a continuum between *dean* and *teen* as beginning with the voiceless /t/, suggesting that they had a more French-like VOT boundary. Thus, those with lower inhibitory skill exhibited more influence from their L2 phonology in their L1. Darcy and colleagues have used a retrieval-induced inhibition task based on the one used by Lev-Ari and Peperkamp (2013) to investigate the relationship between inhibitory control and L2 phonological accuracy. In their study on the L2 phonology of English-speaking learners of Spanish and Spanish-speaking learners of English, Darcy et al. (2016) found that learners with higher inhibitory skill were more accurate at perceiving L2 vowels and more accurate at producing L2 consonants. However, Mora and Darcy (2016) found no relationship between inhibitory control and L2 pronunciation accuracy for learners of English who were L1 Spanish speakers or L1 Spanish-L1 Catalan bilinguals. In a similar study, Darcy and Mora (2016) did find that stronger inhibitory control was related to more accurate perception by L1 Spanish learners of English, although not if they were L1 Spanish-L1 Catalan bilinguals. Ghaffarvand Mokari and Werner (2019) also tested inhibitory skill with a version of the retrieval-induced inhibition task. They reported a positive relationship with inhibitory control and perception for the acquisition of British English vowels by Azerbaijani learners. Inhibitory control was significantly correlated with gain scores, such that those with higher inhibitory skill developed more accurate L2 vowel perception.

Although the connection between inhibitory control and lexical encoding has not previously been investigated, higher inhibitory skill has often been found to be related to less L1-L2 interference in perception and production, and thus it is probable that higher inhibitory skill also is related to less L1-L2 interference in encoding phonolexical representations. Therefore, stronger inhibitory control is hypothesized to correspond to higher accuracy of L2 phonolexical representations in the present study.

#### Attention Control

Attention is an important component in speech learning, since the ability to attend to pertinent information in the speech signal allows an individual to better notice relevant acoustic properties and create new phonetic categories (Francis et al., 2000; Guion and Pederson, 2007). Results of research on the relationship between learners’ attention control and L2 phonological accuracy have been mixed, indicating a positive relationship, a negative relationship, or no relationship between the two (e.g., Kim and Hazan, 2010; Darcy et al., 2014, 2015; Gökgöz-Kurt, 2016; Mora and Darcy, 2016; Safronova, 2016). Gökgöz-Kurt (2016) found a positive relationship between both attention switching and selective attention tasks and gain scores on a test of word-boundary palatalization in English after training. Darcy et al. (2014) tested L1 English-L2 Spanish and L1 Spanish-L2 English bilinguals’ L2 phonological accuracy and their attention-switching ability. The researchers reported that attention control was related to perception and production accuracy, but only for the L1 Spanish-L2 English learners, in that greater attention control was related to more accurate perception. Surprisingly, while greater attention control was also related to higher accuracy for consonants in production, greater accuracy for vowels in production was related to *less* efficient attention control. Safronova (2016) also reported mixed results for the relationship between results on L2 phonological tasks and attention switching for Catalan-Spanish bilinguals. She found that more efficient attention control was related to more perceived distance between L1 and L2 vowels. In contrast, attention control error rate was related to higher accuracy in discrimination, but in the opposite direction as expected. Those learners with a higher error rate in classifying the stimuli according to the correct dimension were those that were more accurate in discrimination. Darcy et al. (2015) found no association between the attention switching scores of Korean learners of English and their performance on a range of L2 phonological tasks. Similarly, Ghaffarvand Mokari and Werner (2019) found no association between attention control, as measured with a Stroop task, and Azerbaijani learners’ improvement on L2 English vowels from high variability phonetic training.

In sum, any relationship between attention control and L2 phonological accuracy is still unclear. The conceptualization of attention control varies greatly in the literature and different tasks are used to test this concept, making it even more difficult to draw definitive conclusions. As for the relationship between attention control and lexical encoding accuracy, it is logical to think that more efficient attention control, operationalized as selective attention or attention switching, would correspond to more accurate lexical representations, since the ability to focus attention on only relevant acoustic cues and efficiently switch attention between those dimensions that matter for L1 sounds versus L2 sounds could aid in acquisition. Nevertheless, this is still an open question that lacks clearly supported predictions based on the mixed results in the aforementioned literature. For the current study, it is tentatively hypothesized that greater selective attention control will correspond to more accurate L2 phonolexical representations.

#### Second Language Vocabulary Size

Another individual difference that may play a role in the development of L2 phonolexical representations is L2 vocabulary size. Research on child language acquisition has found that the development of a vocabulary triggers phonological development. Young children initially store words as more holistic phonological units, but as they add more vocabulary, this leads to more sensitivity to phonological differences between words. In turn, their phonolexical representations are refined in line with their increased phonological awareness (e.g., Metsala and Walley, 1998; Vihman and Croft, 2007). A similar phenomenon has been proposed for L2 learning, in that the creation of an L2 vocabulary is hypothesized to encourage the development of the L2 phonological system. The establishment of increasingly well-defined phonetic categories is in turn thought to feed back into more accurate phonolexical representations (Walley, 2007; Majerus et al., 2008; Bundgaard-Nielsen et al., 2011, 2012).

Several studies to date have examined the effect of vocabulary size on the accuracy of L2 perception and production. Darcy et al. (2015) tested the L1 and L2 productive vocabulary size of Korean learners of English. They found no significant correlations between L1 or L2 vocabulary size and a range of L2 phonological measures. Bundgaard-Nielsen et al. (2011) tested Japanese learners of English studying in Australia on their perceptual assimilation and discrimination of a range of English vowels. The learners did not differ in their years of English study, their length of stay in Australia, the age at which they began learning English, or the age at which they started their immersion experience, but they did differ in vocabulary size. The researchers found that the high vocabulary group consistently had more accurate discrimination of English vowel contrasts than the low vocabulary group. Bundgaard-Nielsen et al. (2012) reported a parallel result for the production of English vowels by Japanese-speaking learners. The vowels produced by the learners in the high vocabulary group as compared to the low vocabulary group were more accurately identified as the intended target by listeners, and vocabulary size as a continuous measure was a significant predictor of average intelligibility, unlike years of English study or length of stay in Australia. Similarly, Mairano and Santiago (2020) reported that vocabulary size correlated moderately with fluency measures and ratings of accentedness. In addition, one study has examined the relationship between L2 vocabulary size and lexical encoding. Llompart (2021b) found that a larger L2 vocabulary was predictive of more accurate phonolexical representations for German learners of English, but only if they were at an advanced level of proficiency.

Overall, these studies suggest that a larger L2 vocabulary leads to a more robust L2 phonological system and more accurate phonolexical representations. Thus, in this study a larger L2 receptive vocabulary size is expected to correspond to higher accuracy in L2 phonolexical encoding.

#### The Current Study

To sum up, individual differences in cognitive abilities and L2 vocabulary knowledge are all likely play a role in the processing and storage of L2 sounds, beyond learners’ accuracy in perception. Greater PSTM may entail holding more detailed representations of L2 sounds in working memory, leading to the creation of more robust long-term representations. Increased inhibitory control may aid in suppressing the L1 phonological system during L2 processing, and stronger attention control may help learners focus attention on L2-relevant dimensions of the speech signal. Finally, a larger L2 vocabulary size may highlight the importance of L2 contrasts through the noticing of continual mismatches with phonological neighbors, leading to the refinement of existing phonolexical representations. Accordingly, the aim of the current study is to determine how well perception, PSTM, inhibitory control, attention control, and L2 vocabulary size each account for L2 lexical encoding accuracy. To investigate this question, our test case is the Spanish /ɾ-r/ (“/tap-trill/”), /ɾ-d/ (“/tap-d/”), /r-d/ (“/trill-d/”), and /f-p/ contrasts. These contrasts have been found to range in discriminability and lexical encoding accuracy for English-speaking learners.

First of all, /tap-trill/ has been found to be accurate in perception but not in lexical encoding. Rose (2010) reported that learners at all proficiency levels were highly accurate at distinguishing the tap and trill in an AXB task, and even naïve English listeners who knew no Spanish were able to discriminate the two phonemes at 80% accuracy. Likewise, Detrixhe (2015) found that intermediate learners were almost at ceiling on a discrimination task and an identification task before going abroad, and Herd (2011) reported that intermediate learners were already quite good at an identification task before training, at 81% accuracy, and improved to 89% accuracy after training. Daidone and Darcy (2014) also found that learners were generally able to perceive the /tap-trill/ distinction in an ABX task; in fact, advanced learners’ accuracy did not significantly differ from that of native speakers. However, Rose (2012) found that both Spanish tap and trill are perceptually assimilated largely to English /ɹ/, which may help explain why Daidone and Darcy (2014) found learners’ lexical encoding accuracy to be low. Learners accepted non-words with the incorrect rhotic in over 70% of cases, such such as accepting ^∗^*quierro* [ki̯ero] as a word, when the real word contains a tap, i.e., *quiero* /ki̯eɾo/ ‘I want.’

Regarding the /tap-d/ distinction, Rose (2010) found that this contrast was significantly less accurate than /tap-trill/ in perception for learners at all levels, ranging from an accuracy of 69.6% for second-semester students to 82.5% for graduate students. Daidone and Darcy (2014) similarly reported that /tap-d/ was less accurate than /tap-trill/, at 64% accuracy for intermediate learners and 82% for advanced learners. The intermediate learners tested by Herd (2011) also struggled to correctly identify tap and /d/ tokens and actually became less accurate after training, going from 70% to 66% accuracy on the identification task, making /tap-d/ the least accurate contrast of the three. Despite the low accuracy of /tap-d/ in perception, Daidone and Darcy (2014) found that it was more accurate in lexical encoding than /tap-trill/. While both intermediate and advanced learners were able to correctly accept tap and /d/ words with an accuracy rate above 90%, they accepted non-words with the incorrect sound at a rate of 65% for the intermediate group and 54% for the advanced group.

The /trill-d/ contrast has been found to be fairly accurate in both perceptual and lexical tasks. This was the most accurate contrast compared to /tap-trill/ and /tap-d/ in the perception results of Daidone and Darcy (2014), with an accuracy rate of 87% for intermediate learners and 94% for advanced learners. Herd (2011) also found that intermediate learners were significantly most accurate at identifying /trill-d/ than /tap-trill/ and /tap-d/, with an accuracy rate of 96% before training and 97% after training. The /trill-d/ contrast was also the most accurate of the three contrasts in lexical encoding in the results of Daidone and Darcy (2014), with word acceptance rates above 80% and non-word erroneous acceptance rates of 39 and 25% for intermediate and advanced learners, respectively.

The /f-p/ contrast served as a control in Daidone and Darcy (2014). Since this contrast also exists in English, it is unsurprising that /f-p/ was significantly more accurate in perception than /tap-trill/, /tap-d/, and /trill-d/ combined for the intermediate learners, and as accurate as these test contrasts for the advanced learners. In lexical encoding, non-word accuracy for /f-p/ was higher than for the test contrasts combined for both groups.

In sum, previous research has found that /trill-d/ is the most accurate in perception, followed by /tap-trill/ and /tap-d/, respectively. The /trill-d/ contrast has been shown to be the most accurate in lexical encoding as well; however, unlike in perception, /tap-d/ has been shown to be more accurate than /tap-trill/. The control contrast /f-p/ has been found to be accurate in both perception and lexical encoding. Given the range in accuracy of discrimination and lexical representations for these contrasts, and the varying relationship between these two constructs, Spanish /tap-trill/, /tap-d/, /trill-d/, and /f-p/ were judged to be a good test case for the relationship between lexical encoding and individual differences in perception, cognitive abilities, and vocabulary size.

## Materials and Methods

This study used a lexical decision task to investigate lexical encoding accuracy, an oddity task to examine perception of the contrasts appearing in the lexical task, a serial non-word recognition task to investigate PSTM, a retrieval-induced inhibition task to measure inhibitory control, a flanker task to investigate attention control, and an X_Lex vocabulary test to estimate Spanish vocabulary size, all described in detail below.

### Lexical Decision Task

A standard auditory lexical decision task was used to provide information on the accuracy of participants’ phonolexical representations. If representations are accurate, learners should accept real words and reject non-words with an incorrect sound. This task has previously been used to examine L2 lexical encoding (e.g., Sebastián-Gallés and Baus, 2005; Darcy et al., 2013).

The lexical decision task used in this study was the same task as employed by Daidone and Darcy (2014). In this task, participants heard a stimulus and indicated whether or not what they heard was a real word of Spanish. Non-words were created by substituting the target phoneme with the other sound in the contrast. For example, the non-word *quierro* /ki̯ero/ was created from the real word *quiero* /ki̯eɾo/ ‘I want’ by substituting the tap for a trill (see Table 1 for more examples). The test contrasts were /tap-trill/, /tap-d/, and /trill-d/; these contrasts were chosen because they were expected to display a range of discriminability and lexical encoding accuracy. In addition, /f-p/ was the control contrast. This contrast was included because an /f-p/ contrast also exists in English, and thus should be relatively easy for learners to discriminate and encode lexically. Furthermore, /f/ and /p/ are similar in place of articulation but differ in manner of articulation, which parallels the test contrasts in that all are similar in place of articulation but differ in manner. Table 1 provides two example words and their non-word counterparts for each condition. The full list of words used in the lexical decision task is available in Supplementary Material.

**TABLE 1:** Example stimuli from lexical decision task.

Condition	Contrast	Stimuli Examples			
		Word		Non-word	
		Orthography	IPA	Orthography	IPA
/tap-trill/	/r-*ɾ/	aburrido ‘bored’	/a.bu.ˈri.do/	aburido	/a.bu.ˈɾi.do/
	/ɾ-*r/	dinero ‘money’	/di.’ne.ɾo/	dinerro	/di.ˈne.ro/
/tap-d/	/ɾ-*d/	cultura ‘culture’	/kul.ˈtu.ɾa/	cultuda	/kul.ˈtu.da/
	/d-*ɾ/	miedo ‘fear’	/ˈmi̯e.do/	miero	/ˈmi̯e.ɾo/
/trill-d/	/r-*d/	ocurre ‘it occurs’	/o.ˈku.re/	ocude	/o.ˈk.ude/
	/d-*r/	estado ‘state’	/es.ˈta.do/	estarro	/es.ˈta.ro/
/f-p/	/f-*p/	jefe ‘boss’	/ˈxe.fe/	jepe	/ˈxe.pe/
	/p-*f/	grupo ‘group’	/ˈgɾu.po/	grufo	/ˈgɾu.fo/

*indicates the sound in the nonword.

In order to find lexical items for the task that would be familiar to learners, an effort was made to choose as many words as possible from the *Beginning Spanish Lexicon*, a database of words from beginner Spanish textbooks (Vitevitch et al., 2012). However, because additional words were needed that contained the target sounds, the L2 Spanish learners who participated in the experiment by Daidone and Darcy (2014) also filled out a word familiarity questionnaire containing all the words from the test and control conditions to gauge their knowledge of the stimuli. This questionnaire revealed that participants in that study were generally very familiar with the words; all contrast conditions averaged 6.3 or above on a 7-point scale (range = 6.32–6.87), with 1 indicating no knowledge of the word and 7 indicating the word was very well known. Words ranged between 2 and 4 syllables, with the target phoneme appearing in intervocalic position as the onset of the 2nd, 3rd, or 4th syllable. All of the stimuli were recorded in a sound booth by two native Spanish speakers: (1) a female speaker from Puerto Rico and (2) a male speaker from Costa Rica. The speakers produced the stimuli with a standard Spanish pronunciation, such that all taps were realized with one occlusion, all trills were realized with at least two occlusions, and /d/ was realized as an approximant [ð̞].

During each trial, a fixation cross appeared in the center of the screen, and participants had 4000 ms to respond from the beginning of the stimulus. The intertrial interval (ITI) was 1000 ms. Different versions of the task were created for right- and left-handed individuals so that a response indicating ‘real word’ always corresponded to a key press with the participant’s dominant hand. Furthermore, two different lists were created so that a word and its non-word equivalent were never heard by the same participant. For example, because the word *quiero* appeared in List 1, the non-word *quierro* appeared in List 2. This resulted in 5 words and 5 non-words for each of the 8 contrasts (see Table 1) in each list, totaling 80 trials. Stimuli were evenly divided between the two speakers for each contrast, and stimuli from the same speaker was used for both the word and its non-word counterpart across lists, e.g., both *quiero* and *quierro* were spoken by the female Puerto Rican speaker. In addition to the test and control stimuli, the same 24 filler words and 24 filler non-words were also included in each list, bringing the total number of trials to 128. The task began with 10 practice trials, during which reminders of what keys to press appeared on the screen (e.g., L = Real, A = Fake), and participants were given feedback on their answers (correct, incorrect, or too slow). Participants needed to score at least 7 out of 10 to precede; otherwise, they repeated the practice trials. This task was administered through a web browser with jsPsych (de Leeuw, 2015) and took participants approximately 6 min to complete.

### Oddity Task

An oddity task containing the contrasts from the lexical tasks was constructed in order to investigate the ease of discriminability of these sounds. This task was chosen instead of other common perception tasks, such as AX or ABX, because it is a cognitively more demanding task (Strange and Shafer, 2008), and therefore was less likely to result in ceiling effects for the easier contrasts. In addition, because the chance level is lower in an oddity task (25%) compared to an AX or ABX task (50%), it was expected to yield more variation in scores.

In this task, participants heard three stimuli in a row and were instructed to choose which of the three was different, or alternately, that they were all the same. For example, if they heard *lefo-lepo-lefo*, the participant was expected to indicate that the second stimulus was different. The conditions were the same as those appearing in the lexical task, that is, /tap-trill/, /tap-d/, /trill-d/, and /f-p/. Filler trials that represented other contrasts were also included. All stimuli were disyllabic Spanish non-words. Stimuli were also non-words in English. Three non-words pairs per contrast were created with the target consonants always appearing as the onset of the second syllable, such as *terro-tedo* /tero/-/tedo/. The full list of stimuli is available in Supplementary Material. Stimuli were recorded by a female simultaneous Spanish-English bilingual who spoke Mexican Spanish, a male Costa Rican Spanish speaker, and a female Puerto Rican Spanish speaker. The Costa Rican speaker and the Puerto Rican speaker were the same speakers that were recorded for the lexical task. Three different Spanish speakers were recorded because using different voices reduces participants’ reliance on purely episodic memory to complete the task (Ramus et al., 2010); instead, participants must categorize the sounds at a phonological level to compare across speakers. Only tokens with a standard Spanish pronunciation were selected for the task; for example, all examples of the trill had at least two clear occlusions.

For every trial, each token was spoken by a different speaker, always in the same order: (1) the female simultaneous Spanish-English bilingual who spoke Mexican Spanish, (2) the male Costa Rican Spanish speaker, (3) the female Puerto Rican Spanish speaker. Participants indicated their response by clicking on one of three robots in a row on the screen according to which one “said” something different, or by clicking on the X following the robots to indicate that all the words were the same.

Each of the stimuli pairs appeared once in the 8 possible combinations of orders (AAA, BBB, ABB, BAA, ABA, BAB, AAB, BBA). For example, the *nera-nerra* stimuli pair appeared once as *nera-nera-nera* (AAA), once as *nerra-nerra-nerra* (BBB), once in the order *nera-nerra-nerra* (ABB), etc. This resulted in 24 trials per contrast and 96 test and control trials total. In addition, 48 filler trials were included, bringing the total number of trials to 144. These filler pairs also all appeared in the 8 possible combination of orders. The interstimulus interval (ISI) in each trial was 400 ms, the ITI was 500 ms, and the timeout for the trials was set to 6500 ms from the start of the trial. Participants also completed 8 training trials in order to familiarize them with the task. Participants needed to correctly respond to at least 6 out of 8 of the practice trials to precede to the actual task, or else they repeated the practice trials. The task lasted approximately 10 min, with one break in the middle, and was administered through a web browser with jsPsych. Each block contained an equal number of trials per condition, and trials were randomized within each block.

### Phonological Short-Term Memory Task

A serial non-word recognition task adapted from the one used in Zahler and Lord (in press) was employed to examine PSTM. Following Cerviño-Povedano and Mora (2015), a non-word recognition task was chosen over a non-word repetition task because the latter involves production of the stimuli, and participants’ ability to articulate the Russian sounds would likely have differed. Furthermore, serial recognition is less affected by the lexical status of the stimuli than serial recall, which suggests that a recognition task is a better indicator of short-term memory ability rather than knowledge of representations stored in long-term memory (O’Brien et al., 2007).

In this task, participants heard sequences of Russian stimuli and had to decide if the two sequences were in the same order or a different order. The task became progressively harder as the two sequences that participants needed to compare became longer, starting at four stimuli in a row for each sequence and ending at seven stimuli in a row. The stimuli were CVC sequences spoken by a female native speaker of Russian (see Supplementary Material). Although some of the Russian stimuli were real words in Russian, all of the stimuli in this task will be referred to as non-words because they were all unknown from the participants’ point of view.

Stimuli were organized into sequences. Non-words within a sequence were separated by 300 ms pauses, and the two sequences in a trial were separated by a 2000 ms pause. For the different-order trials, two stimuli in the middle of the sequence were always switched (e.g., ABCDE vs. ACBDE; ABCDE vs. ABDCE), while the first and last stimulus were always in the same position. No minimal pairs were used within a sequence and adjacent stimuli did not share any phonemes. After both sequences had finished playing, participants were shown a screen reminding them of the key presses for ‘same’ and ‘different’ and given 3000 ms to respond. The ITI was 1000 ms. Participants completed 8 trials for each of the sequence lengths (4, 5, 6, and 7 non-words), for 32 trials in total. Trials were blocked by sequence length, starting with sequences of 4 and ending with sequences of 7. Before beginning the actual task, participants had to correctly respond to 3 out of the 4 practice trials with a sequence length of 4 non-words; the practice repeated as necessary. The PSTM task was administered using jsPsych and took 7 min to complete.

### Inhibitory Control Task

The task employed to investigate inhibition was a retrieval-induced inhibition task like the one used in Lev-Ari and Peperkamp (2013) and Darcy et al. (2016). This task was chosen to investigate inhibitory control because other tasks often used to measure inhibition, such as the Stroop task, can also be considered measures of selective attention to external stimuli, and a separate task was used in the current study for that measure.

This task consisted of three phases: memorization, practice, and test. Participants first were instructed to memorize the 18 words. The words were individually presented on the screen with their category (e.g., “FRUITS – apple”) for 5 s. In the practice phase, participants practiced half of the words from two of the categories, each three times. The categories and words that were practiced were randomized across participants. In order to practice the words, participants were presented with a category and the first letter of a word (e.g., “FRUITS-a”) with a blank textbox below. They then needed to type the relevant word into the textbox. In the test phase, participants were presented with a word (e.g., “apple”) and had to indicate whether each word shown on the screen was a word that they have learned in the memorization phase. Each trial was preceded by a fixation cross in the center of the screen for 1500 ms, and once the word appeared participants had 3000 ms to respond.

Stimuli were 6 words in each of 3 categories – fruits, occupations, and animals – for a total of 18 words (see Supplementary Material). The words were assigned into three possible conditions: practiced, inhibited, and control. Practiced items were memorized and then practiced by the participant. Inhibited items were memorized as well, but they were not practiced by the participant. However, they belonged to the same semantic category as other words that were practiced. Control items were memorized by the participant but were not subsequently practiced by them, and none of the words in that specific category were practiced. For example, if *fruits* was the control category for a participant, they would then memorize and practice half the words from each of the *occupations* and *animals* categories.

By having participants practice only some of the words that they memorized, this task led participants to inhibit the other learned items from those categories, because retrieving words from a semantic category necessitates the suppression of other words in that category. For example, if a participant memorized “nurse” and “dentist” but then only practiced “nurse”, the word “dentist” should be inhibited and thus take more time to retrieve and respond to. In contrast, a word in the *animals* category like “wolf” should not have been inhibited and therefore be faster to respond to than “dentist”, while “nurse” should elicit an even faster RT since it was practiced and therefore more strongly activated.

All of the 18 words they had initially memorized were included in the test phase, as well as 18 distractor words from the same semantic categories, resulting in an equal number of ‘yes’ and ‘no’ correct answers. Two versions of the task were created so that a ‘yes’ response corresponded to a key press with the participant’s dominant hand for both right- and left-handed individuals. This 6-min task was administered through a web browser with jsPsych.

### Attention Control Task

A flanker task, a non-verbal test of selective attention, was used to investigate attention control (Eriksen, 1995). The choice to use a non-verbal task rather than a speech-based attention-switching task was made in order to ensure as much as possible that the attention control task was testing a different construct than the verbal retrieval-induced inhibition task.

In this task, participants decided which way the center arrow was facing out of a group of five arrows. In congruent trials, all arrows faced the same direction (e.g., →→→→→), while in incongruent trials the middle arrow faced the opposite duration of the flanking arrows (e.g., →→←→→). Participants’ ability to select relevant information (the center arrow) and ignore distracting information (the flanking arrows) tested their spatial selective attention ability, which is operationalized as the difference between reaction times to congruent and incongruent trials (Bugg and Crump, 2012). This is also known as the conflict effect or executive control (Fan et al., 2002). The smaller the difference in reaction times to congruent and incongruent trials, the better able the participant is to focus their attention on the relevant dimension.

Each trial was preceded by a fixation cross in the middle of the screen for 400 ms, after which time the arrows appeared. Participants pressed the right arrow key to indicate a right-facing arrow in the center, and the left arrow key to indicate a left-facing arrow in the center. They had 1700 ms to respond, after which point there was a 400 ms pause before the next trial. Participants first completed a training phase with feedback. In the following test phase, the 4 possible types of trials (right-facing congruent, right-facing incongruent, left-facing congruent, and left-facing incongruent) were each repeated 20 times, for a total of 80 trials. The flanker task was run through a web browser using jsPsych, and it lasted approximately 3 min.

### Spanish Vocabulary Test

The X_Lex vocabulary test was used to estimate participants’ receptive Spanish vocabulary size (Meara, 2005). This task was chosen because it tests words in the 0–5,000 frequency range, and it was anticipated that targeting this frequency range would capture variation in learners’ knowledge without producing floor effects. In this task, participants were presented with a randomized sampling of 100 Spanish words which were evenly distributed among the 1K, 2K, 3K, 4K, and 5K frequency bands. The test also included 20 plausible Spanish non-words to correct for any bias toward answering yes to unknown words. Participants indicated whether or not they knew a word shown on the screen by clicking on the happy face for ‘yes’ and the sad face for ‘no’. The vocabulary task took around 5 min for participants to complete.

### Participants

Participants in this study were English-speaking learners of Spanish^1^. These learners were either undergraduate Spanish majors and minors enrolled in a fifth-semester or higher-level Spanish course or graduate students that had taken graduate courses in Spanish. Most of the graduate students were teaching Spanish and studying Hispanic linguistics or Hispanic literatures and cultures. They had all grown up in monolingual households in which only English was spoken.

In total, 42 L2 learners of Spanish were tested. However, three participants were excluded from all analyses for various reasons (see Daidone, 2020). This resulted in a final count of 39 L2 learners for inclusion in the analyses. The demographic info for all remaining participants is available in Table 2. Participants were also excluded on a task-by-task basis when necessary. These exclusions are discussed under the analysis and results section for each task.

**TABLE 2:** Demographic information for participants.

	L1 English-L2 Spanish Learners *N* = *39*
Age at testing (years)	22.4 (3.8)
Age of onset for L2 learning	13.1 (2.5)
Residence in a Spanish-speaking country (months)	2.5 (6.2)
Self-rated L2 speaking ability (0–6)	3.9 (1.7)
Self-rated L2 listening ability (0–6)	4.2 (1.5)
Self-rated L2 reading ability (0–6)	4.5 (1.3)
Self-rated L2 writing ability (0–6)	4.4 (1.5)
Gender	27 female
Handedness	3 left-handed

*Means are given for rows 1–8, with standard deviations in parentheses. Counts are given for rows 9 and 10.*

### General Procedure

After viewing the study information sheet and consenting to take part in the study, participants completed a bilateral hearing screening. All participants needed to pass the hearing screening in order for their data to be included in the analyses. Participants next completed the lexical decision task, oddity task, and a forced choice lexical decision task that is not discussed in the current study (see Daidone, 2020, for more details). They then moved onto the serial non-word recognition task, flanker task, retrieval-induced inhibition task, and X_Lex vocabulary test. Lastly, they completed a language background questionnaire, which also included a word familiarity section for the words used in the lexical decision task. For the tasks that presented auditory stimuli, participants wore Sennheiser HD 515 over-ear headphones. The entire experiment lasted 65–75 min and individuals were paid $15 for participating.

## Results

### Results and Analyses by Task

#### Lexical Decision Task Analysis and Results

The lexical decision task directly assessed the accuracy of participants’ lexical representations for words containing the Spanish contrasts we examine. The ability to reject a non-word is contingent on its word counterpart being accurately represented in the lexical entry. We use *d’* (“d-prime”) scores as a bias-free measure of perceptual sensitivity to the lexical status of non-words; thus, a higher *d’* indicates more accurate lexical representations for that contrast. Generally, *d*’ scores below 0.75 can be interpreted as a lack of discrimination sensitivity. Scores from 0.75 to 3.0 show increasing discrimination sensitivity, and scores above 3.0 show very strong discrimination sensitivity.

Data for the lexical task were not saved for two participants due to a coding error. Trials with timeouts were excluded from the analysis. Participants needed to have responses to minimally 95% of trials in order to be included (i.e., 6 or fewer timeouts). No learner had to be excluded for timeouts.

**FIGURE 1:**
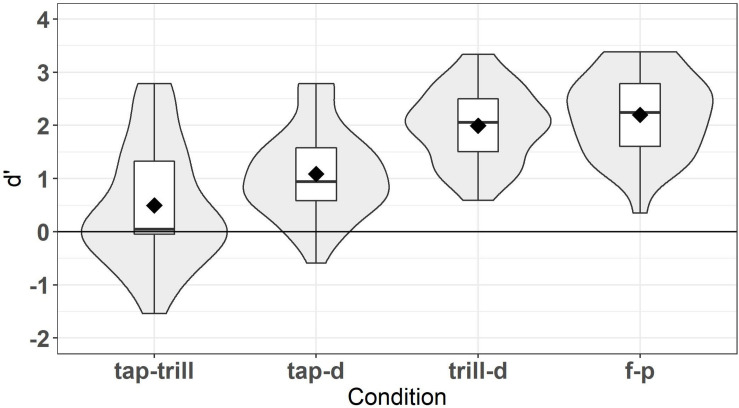
*d’* scores for lexical decision task.

Despite the fact that the words in the lexical tasks were chosen in order to be familiar to L2 learners, it is likely that some words were unknown, and therefore a response on these trials would not be a reliable reflection of learners’ phonolexical knowledge. Because of this, learners’ responses on the word familiarity section of the background questionnaire were taken into account. Vocabulary knowledge was evaluated on an individual basis for each participant. For a trial to be included, the participant had to have chosen one of the three highest options on the 6-point word familiarity scale for that word. Vocabulary knowledge was considered for non-word trials as well. The inclusion of non-word trials was evaluated based on the participant’s familiarity with the corresponding real word, with the exception of the filler condition where non-words were not based on real words. For example, if the word *desarrollo* ‘development’ was not known, the non-word counterpart trial *desadollo* was excluded from the analysis. If participants had less than half of word or non-word trials remaining in a condition, their results were excluded from the analysis. Two participants’ results were excluded for remaining with less than half of the non-word trials in the /trill-d/ condition and the /f-p/ condition, respectively. The final number of L2 learners who were included in the lexical decision task analyses was 35, with an almost even split between those who completed List 1 (17 participants) and those who completed List 2 (18 participants). Figure 1 displays the *d’* scores for each condition, excluding trials with timeouts and unknown words. Diamonds represent mean values, and violin plots around the boxplots show the distribution of scores.

Overall, as shown in Figure 1, the results of the lexical task show that learners had the lowest scores for the /tap-trill/ condition, followed by the /tap-d/ condition, while the /trill-d/ and /f-p/ conditions were both the most accurate. This suggests that lexical representations are overall most accurate for the trill-d and f-p contrasts, and least accurate for the tap-trill contrast. Analyses of Variance (ANOVAs) were conducted to examine the effects of condition (contrast) and list. A two-way mixed ANOVA was run with *d’* score as the dependent variable, *condition* (/tap-trill/, /tap-d/, /trill-d/, /f-p/) as the within-subjects independent variable, and *list* (1 vs. 2) as the between-subjects independent variable. The ANOVA test and tests for checking the assumptions of an ANOVA were conducted in R using the *rstatix* package v.0.3.1 (Kassambara, 2019). All assumptions for the ANOVA were met regarding normality, sphericity, and homogeneity of variances and covariances. The Bonferroni correction method was used to adjust *p-*values for multiple comparisons in *post hoc* tests, which were conducted with the built-in *stats* package in R version 4.0.2 (R Core Team, 2020). The ANOVA revealed that there was a significant interaction between condition and list, *F*(3, 99) = 7.654, *p* < 0.001. Condition was significant for both lists (*p* < 0.001), such that within each list, conditions differed from each other, with some slight differences. For List 1, only /trill-d/ vs. /f-p/ (*p* = 0.734) and /tap-trill/ vs. /tap-d/ (*p* = 1) did not differ from each other, while in List 2, only /trill-d/ vs. /f-p/ (*p* = 1) and /tap-d/ vs. /f-p/ (*p* = 0.216) did not differ from each other. However, *d’* scores did not differ between lists for any of the conditions (all *p* > 0.1), nor was there a main effect of list (*p* = 0.568). For this reason, it was judged appropriate to combine scores across the two lists for the individual differences analyses. The main effect of condition was significant, *F*(3, 99) = 65.412, *p* < 0.001. When lists were combined, all conditions were significantly different from each other (all *p* < 0.001) with one exception; performance on /trill-d/ was not different from /f-p/ (*p* = 1). This task largely replicated the results of Daidone and Darcy (2014) and yielded substantial variation in scores for the L2 learners, making it suitable for use in the individual differences analyses.

#### Oddity Task Analysis and Results

**FIGURE 2:**
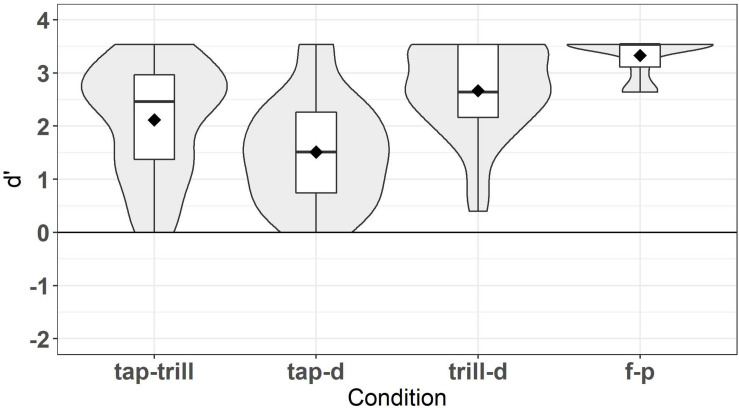
*d’* scores for oddity task.

The oddity task was used to examine participants’ perception ability for the Spanish contrasts that appeared in the lexical task (/tap-trill/, /tap-d/, /trill-d/, and /f-p/). For each contrast, *d’* scores were computed rather than accuracy because the learners showed a strong bias in the /tap-d/ condition and to a lesser extent in the /tap-trill/ condition toward choosing that the trials were the same, and *d’* provides a bias-free measure of perceptual sensitivity. The *d’* scores were calculated by grouping trials as same (AAA, BBB) or different (AAB, BBA, ABA, BAB, ABB, BAA). If participants recognized that one of the sounds was different, even if they did not correctly identify which sound was different, this counted as a hit, whereas if they chose any of the stimuli as different when they were all the same, this was counted as a false alarm. Trials with timeouts were excluded. Participants could not have timeouts on more than 5% of trials (i.e., 7 timeouts) in order to be included; no participant had timeouts on more than 2 trials. Therefore, all 39 learners were included in the analysis. Results of the *d’* analysis are illustrated in Figure 2, indicating that learners were highly accurate on the /f-p/ condition and less accurate on the /trill-d/ condition, followed by the /tap-trill/ condition and the /tap-d/ condition, respectively. This result is expected based on the findings of Daidone and Darcy (2014). Notably, the order of accuracy is not the same as for lexical decision, just as Daidone and Darcy found.

Inferential statistics confirmed that learners’ performance differed by contrast. A non-parametric Friedman test was run in R using the *rstatix* package v.0.3.1 (Kassambara, 2019) because the data violated the assumptions for a repeated measures ANOVA. Specifically, the results for the /tap-trill/, /trill-d/, and /f-p/ conditions were not normally distributed, and the assumption of sphericity was also violated. The Friedman test was conducted with *d’ score* as the dependent variable and *condition* (/tap-trill/, /tap-d/, /trill-d/, /f-p/) as the independent variable. *Post hoc* tests were adjusted for multiple comparisons with the Bonferroni correction method. Results revealed that *d’* scores were significantly different across conditions, χ^2^(3) = 69.846, *p* < 0.001. Pairwise Wilcoxon signed-rank tests found that all conditions were significantly different from each other (all *p* < 0.05). Therefore, these contrasts varied in discriminability. Learners also showed substantial variation within each condition, at least for /tap-trill/, /tap-d/, and /trill-d/, making these scores acceptable for individual differences analyses.

#### Phonological Short-Term Memory Task Analysis and Results

The PSTM task examined how well participants were able to hold increasingly longer sequences of sounds in memory and compare them. The more accurate they were at correctly identifying if these sequences were the same or different, the greater their PSTM. In order to analyze the PSTM task, the response to each test trial was coded as 1 or 0. If the participant correctly identified the paired sequences of Russian CVC non-words as being in the same order or a different order, they received a 1 for that trial, and if they were incorrect or timed out, they received a 0. No participant had more than one timeout. Participants earned a score out of 8 for each sequence length (4, 5, 6, or 7 non-words) as the block for each sequence length contained 8 trials. In accordance with Zahler and Lord (in press), scores were then weighted by the length of the sequences, such that the score for each length block was multiplied by the length itself (4, 5, 6, or 7). For example, a participant who correctly responded to 6 trials of length 4 received a score of 6 × 4 = 24 for those trials. This resulted in a total possible weighted score of 176 [(8 × 4) + (8 × 5) + (8 × 6) + (8 × 7) = 176]. Scores ranged from 78 to 151 (*M* = 114, SD = 18).

#### Inhibition Task Analysis and Results

The retrieval-induced inhibition task examined participants’ inhibitory skill by testing how much slower they responded to memorized words that were inhibited due to the effect of having retrieved semantically related words during the practice phase. A slower reaction time (RT) to these items indicated more inhibition, and thus higher inhibitory control. Inhibitory skill was calculated using median RTs in accordance with the technique reported by Lev-Ari and Peperkamp (2013). First, the median RT was determined for each participant for each of the three conditions in the test phase (practiced, inhibited, and control). The practiced items were those words that also appeared during the practice phase, the inhibited items were those that came from a practiced category, but did not form part of the practice phase, and control items were those that came from a category that was not part of the practice phase at all. For example, if a participant had to type the words *engineer, nurse, carpenter, grape, cherry*, and *orange* during the practice phase, then the RTs for the recognition of these words in the test phase fell under the practiced condition, the other words under the categories *occupations* and *fruits* were part of the inhibited condition, and all words in the *animals* category formed part of the control condition.

Following Darcy et al. (2016), if participants missed all instances of two or more words during the practice phase of the task, they were excluded. Four L2 learners were excluded for this reason, resulting in a total of 35 included participants. For the test part of the task, trials with an RT beyond 2 SD in either direction from the average for that participant were removed. No participant had more than two trials removed for this reason.

The data exhibited extreme outliers and violated the assumptions of sphericity and normality for all conditions for a repeated measures ANOVA. Thus, a non-parametric Friedman test was run in order to examine if *median RT* (dependent variable) differed by *condition* (practiced, inhibited, control). The Friedman test revealed that RT was significantly different across conditions, χ^2^(2) = 12.189, *p* = 0.002. Pairwise Wilcoxon signed-rank tests found that, as hypothesized, inhibited items were responded to more slowly than practiced items (Bonferroni adjusted *p* = 0.003). Although median RTs to control items (808 ms) were numerically slower than practiced items (761 ms) and faster than inhibited items (856 ms), median RTs to control items did not significantly differ from inhibited items (adjusted *p* = 0.235) or from practiced items (adjusted *p* = 0.184). An inhibition score for each participant was calculated by dividing the median RT for inhibited items by the median RT for control items; higher values indicate greater inhibitory skill (Lev-Ari and Peperkamp, 2013). Inhibition scores ranged from 0.71 to 1.65 (*M* = 1.07, SD = 0.19).

#### Attention Control Task Analysis and Results

Participants’ ability to selectively attend to the center arrow while ignoring the surrounding arrows (in other words, to respond equally as quickly when the surrounding arrows did not match the direction of the center arrow) served as the measure of attention control. Two L2 learners were excluded from the analysis because they had timeouts on more than 5% of trials, leaving a total of 37 participants. The mean and SD for each participant was calculated, and RTs beyond two SDs from the mean in either direction were excluded. All participants were left with at least 36 trials out of 40 in each condition (i.e., congruent and incongruent). In order to investigate whether there was a significant difference in RTs between congruent and incongruent trials, a two-tailed paired samples t-test was run. Results showed that there was a significant effect of condition, such that congruent trials (*M* = 441 ms) were responded to faster on average than incongruent trials (*M* = 467 ms), *t*(36) = −8.401, *p* < 0.001. For each participant, the mean RT for congruent and incongruent trials was derived and the RT differences between the congruent and incongruent trials (congruent average RT - incongruent average RT) was calculated for the measure of selective attention. Scores closer to zero designate better selective attention, that is, less of a reaction time difference between the congruent and incongruent conditions, although in some cases participants’ scores were unexpectedly negative, indicating faster responses to incongruent trials on average. L2 learners exhibited a range of scores, with a min of −23 ms and a max of 69 ms (*M* = 27 ms, SD = 19 ms).

#### Vocabulary Test Analysis and Results

Learners’ ability to recognize real Spanish words at different frequency bands and reject non-words was used to estimate their L2 vocabulary size, with more acceptance of words and rejection of non-words indicating more robust vocabulary knowledge. The measure of vocabulary size was their adjusted vocabulary scores out of 5000 generated by the X_Lex vocabulary test (Meara, 2005). According to the X_Lex manual, these adjusted scores were calculated by subtracting the overall false alarm rate from the hit rate for each frequency band. For example, if a participant scored 20/20 on each of the 5 frequency bands (1K, 2K, 3K, 4K, and 5K), but responded ‘yes’ to 3 non-words, then their adjusted score for each frequency band would be 17/20. If the number of false alarms was higher than the hit rate, this was coded as a score of 0 for that frequency band. Accuracy was averaged across the frequency bands (0.85 for this example participant whose adjusted score was 17/20 for each frequency band) and multiplied by 5000 to result in a score out of 5000 (in the example participant’s case, 4250). All 39 participants were included in the analysis. Participants’ vocabulary scores ranged from 400 to 4850 (*M* = 2792, SD = 1110).

### Individual Differences Analyses

In order to examine how the individual differences measures (perception, PSTM, inhibition, attention, and vocabulary size) contributed to lexical encoding accuracy, a linear regression analysis was run on the complete dataset, and individual regression analyses were run for each contrast. For all of the analyses, the individual differences measures were converted into z-scores, and lexical decision *d’* scores were used for the dependent variable. The individual differences measures did not exhibit high levels of collinearity; the variance inflation factor (VIF) for variables across all analyses was less than 2, whereas problematic collinearity would be indicated by values of 5 or higher (Heiberger and Holland, 2004, 243). For each analysis, the oddity perception measure always matched the condition used for the lexical measure; for example, in the analysis examining the impact of individual differences on the /tap-trill/ condition in the lexical decision task, only performance on the /tap-trill/ condition was included in the oddity z-score calculation.

While we originally attempted to fit a linear mixed effects model with random intercepts for participants, this resulted in a singular fit with variance and standard deviation of the random intercept both estimated at 0. Thus, we decided to run a linear regression model with fixed effects only. This analysis was run in R with the *stats* package version 3.6.2 (R Core Team, 2020), with lexical decision scores as the dependent variable and all individual differences measures (oddity, PSTM, inhibitory control, attention control, and vocabulary size) and their interactions with condition (/tap-trill/, /tap-d/, /trill-d/, and /f-p/), as well as condition alone, as the independent variables.

For the overall analysis, the multiple regression was significant, *F*(23, 96) = 8.446, *p* < 0.001. As Table 3 illustrates, vocabulary score was the only significant predictor of overall lexical decision performance, with greater vocabulary size predicting more accurate lexical encoding. Additionally, the /tap-trill/ and /tap-d/ conditions significantly differed from the baseline /f-p/ condition, which replicates the results of the ANOVA on the lexical decision results in section 3.1.1. None of the other main effects or interactions were significant.

**TABLE 3:** Summary of regression analysis for lexical decision, all conditions.

Predictor	B	B 95% CI	Std Error B	*t*-Value	*p*	
(Intercept)	2.07	[1.80, 2.34]	0.137	15.093	< 0.001	***
Oddity	0.17	[−0.17, 0.50]	0.169	0.983	0.328	
PSTM	0.18	[−0.10, 0.46]	0.145	1.254	0.213	
Inhibition	0.07	[−0.21, 0.36]	0.147	0.508	0.613	
Flanker	0.14	[−0.17, 0.45]	0.157	0.908	0.366	
Vocab	0.46	[0.16, 0.75]	0.150	3.054	0.003	**
/tap-d/ condition	–1.10	[−1.47, −0.72]	0.190	–5.801	< 0.001	***
/tap-trill/ condition	–1.62	[−1.99, −1.24]	0.189	–8.533	< 0.001	***
/trill-d/ condition	–0.21	[−0.58, 0.17]	0.191	–1.089	0.279	
Oddity x/tap-d/	–0.18	[−0.64, 0.28]	0.236	–0.762	0.448	
Oddity x/tap-trill/	0.11	[−0.36, 0.58]	0.240	0.461	0.646	
Oddity x/trill-d/	–0.09	[−0.57, 0.39]	0.245	–0.36	0.720	
PSTM x/tap-d/	–0.10	[−0.49, 0.30]	0.202	–0.472	0.638	
PSTM x/tap-trill/	0.36	[−0.05, 0.77]	0.209	1.731	0.087	
PSTM x/trill-d/	–0.10	[−0.49, 0.30]	0.201	–0.472	0.638	
Inhibition x/tap-d/	–0.28	[−0.69, 0.14]	0.212	–1.297	0.198	
Inhibition x/tap-trill/	–0.05	[−0.45, 0.36]	0.209	–0.216	0.830	
Inhibition x/trill-d/	–0.03	[−0.44, 0.38]	0.209	–0.135	0.893	
Flanker x/tap-d/	–0.12	[−0.57, 0.32]	0.227	–0.541	0.590	
Flanker x/tap-trill/	0.15	[−0.28, 0.59]	0.221	0.686	0.495	
Flanker x/trill-d/	–0.01	[−0.45, 0.42]	0.223	–0.061	0.952	
Vocab x/tap-d/	0.15	[−0.27, 0.56]	0.214	0.681	0.498	
Vocab x/tap-trill/	–0.01	[−0.48, 0.47]	0.243	–0.035	0.972	
Vocab x/trill-d/	0.08	[−0.33, 0.49]	0.210	0.374	0.709	
*Overall Fit*	*R*^2^ = *0.669 p* = < 0.001***					

B = unstandardized regression weight. CI = confidence interval. **p < 0.01, ***p < 0.001.

The multiple linear regression analyses were run on the data for each contrast in R using the *stats* package, with tables created in part with the *apaTables* package v.2.0.5 (Stanley, 2018). All confidence intervals were calculated with the bootstrap method described in Algina et al. (2008) using the *apa.reg.boot.table* function, as recommended for smaller sample sizes and data that violate the assumptions of normality or homogeneity of variances. Only 30 complete cases remained after excluding participants with missing data points.

The multiple regression analyses for /tap-trill/ (*F*(5, 24) = 4.79, *p* = 0.004), /tap-d/ (*F*(5, 24) = 5.449, *p* = 0.002), and /trill-d/ (*F*(5, 24) = 4.908, *p* = 0.003) were significant (shown in Tables 4–6, respectively), while the regression for /f-p/ was not (*F*(5, 24) = 1.858, *p* = 0.140). This indicates that for all contrasts except /f-p/, lexical performance is explained in part by a combination of the other factors. We now consider each contrast in turn. As seen in Table 4, PSTM and vocabulary size were significant predictors of lexical decision scores in the /tap-trill/ condition. They each accounted for a similar amount of variance in lexical decision scores (ΔR^2^), approximately 26% for PSTM and 22% for vocabulary size.

**TABLE 4:** Summary of regression analysis for lexical decision, /tap-trill/ condition.

Predictor	B	B 95% CI	Std Error B	*t*-value	ΔR^2^	ΔR^2^ 95% CI	*p*	
(Intercept)	0.46	[0.15, 0.78]	0.163	2.795	NA	NA	0.010	*
Oddity	0.15	[−0.29, 0.56]	0.206	0.712	0.01	[0.00, 0.11]	0.483	
PSTM	0.62	[0.26, 0.94]	0.176	3.513	0.26	[0.03, 0.45]	0.002	**
Inhibition	–0.02	[−0.46, 0.26]	0.190	–0.085	0.00	[0.00, 0.08]	0.933	
Flanker	0.38	[−0.14, 0.77]	0.205	1.880	0.07	[0.00, 0.24]	0.072	
Vocab	0.61	[0.19, 1.12]	0.189	3.244	0.22	[0.03, 0.43]	0.003	**
*Overall Fit*	*R*^2^ = *0.499, 95% CI[0.31,0.77], p* = 0.004**							

B = unstandardized regression weight. ΔR^2^ = the change in R^2^ when the variable is removed. Numbers in brackets indicate the lower and upper limits of a bootstrapped 95% confidence interval. * p < 0.05, **p < 0.01.

**TABLE 5:** Summary of regression analysis for lexical decision, /trill-d/ condition.

Predictor	B	B 95% CI	Std Error B	t-value	ΔR^2^	ΔR^2^ 95% CI	*p*	
(Intercept)	1.86	[1.66, 2.07]	0.099	18.892	NA	NA	< 0.001	***
Oddity	0.08	[−0.18, 0.33]	0.132	0.590	0.01	[0.00, 0.10]	0.561	
PSTM	0.09	[−0.12, 0.30]	0.104	0.831	0.01	[0.00, 0.15]	0.414	
Inhibition	0.05	[−0.25, 0.24]	0.110	0.425	0.00	[0.00, 0.10]	0.675	
Flanker	0.13	[−0.19, 0.45]	0.117	1.106	0.03	[0.00, 0.24]	0.280	
Vocab	0.54	[0.31, 0.75]	0.109	4.922	0.50	[0.16, 0.69]	< 0.001	***
*Overall Fit*	*R*^2^ = *0.506, 95% CI[0.32,0.82], p* = 0.003**							

B = unstandardized regression weight. ΔR^2^ = the change in R^2^ when the variable is removed. Numbers in brackets indicate the lower and upper limits of a bootstrapped 95% confidence interval. **p < 0.01, ***p < 0.001.

**TABLE 6:** Summary of regression analysis for lexical decision, /tap-d/ condition.

Predictor	B	B 95% CI	Std Error B	*t*-value	ΔR^2^	ΔR^2^ 95% CI	*p*	
(Intercept)	0.97	[0.70, 1.20]	0.120	8.046	NA	NA	< 0.001	***
Oddity	0.15	[−0.22, 0.46]	0.157	0.988	0.02	[0.00, 0.15]	0.333	
PSTM	0.03	[−0.25, 0.30]	0.140	0.225	0.00	[0.00, 0.08]	0.824	
Inhibition	–0.20	[−0.44, 0.01]	0.136	–1.453	0.04	[0.00, 0.17]	0.159	
Flanker	0.00	[−0.22, 0.28]	0.144	0.017	0.00	[0.00, 0.05]	0.987	
Vocab	0.49	[0.16, 0.91]	0.176	2.783	0.15	[0.01, 0.41]	0.010	*
*Overall Fit*	*R*^2^ = *0.532, 95% CI[0.39,0.79], p* = 0.002**							

B = unstandardized regression weight. ΔR^2^ = the change in R^2^ when the variable is removed. Numbers in brackets indicate the lower and upper limits of a bootstrapped 95% confidence interval. *p < 0.05, **p < 0.01, ***p < 0.001.

Table 6 displays the summary of the /tap-d/ analysis, showing that only vocabulary scores were a significant predictor of performance on the lexical decision task in this condition, explaining approximately 15% of the variance in scores.

Table 5 shows the results of the /trill-d/ analysis. Similar to the /tap-d/ analysis, only vocabulary size was a significant predictor of lexical decision performance in this condition, although it explained a larger portion of the variance in this analysis, around 50%.

In sum, vocabulary size was a significant predictor of lexical encoding accuracy in the overall analysis and for three of the four contrasts investigated, specifically /tap-trill/, /tap-d/, and /trill-d/, while PSTM was only significant for the individual /tap-trill/ analysis. Learners’ scores in the oddity, flanker, and inhibition tasks were not significant predictors of lexical decision performance for any contrast.

## Discussion

While perception was predicted to have a large effect on lexical encoding accuracy, surprisingly there was no effect for any of the analyses. Most models of L2 speech acquisition implicitly or explicitly propose a direct link between perception ability and the accuracy of phonological representations in the lexicon ([SLM] Flege, 1995; [PAM-L2] Best and Tyler, 2007; [L2LP] van Leussen and Escudero, 2015)^2^. The lack of an effect for perception ability in the current study seems to contradict this assumption. Instead, the factor with the largest impact on L2 lexical encoding for most contrasts was revealed to be L2 vocabulary size. These results support the premise of a lexicon-first model like NLM-e, which proposes that learning phonological neighbors aids in the formation of phonetic categories, which in turn leads to refinement in the phonetic detail of existing phonolexical representations (Kuhl et al., 2008), as has been found for young children learning their L1 (see Stoel-Gammon, 2011, for a review). This idea is also touched on by Best and Tyler (2007) in their discussion of PAM-L2, in which they assert that the learning of many minimal pairs would exert pressure on learners’ phonological system to begin to distinguish those sounds. This suggests that the acquisition of more and more phonologically similar words forces learners’ phonological system to create more detailed representations in order for them to be differentiated.

Thus, the accuracy of learners’ representations appears to stem more from properties of their lexicon over their perception abilities. However, it is also important to note that the Spanish contrasts examined in the current study were not particularly difficult for the English learners, with *d’* scores in the oddity perception task all averaging above 1.5 across conditions. It may be that beyond a certain threshold of accuracy, differences in perception ability no longer have an appreciable difference on lexical encoding accuracy, whereas they would be very important for more difficult contrasts. This hypothesis is in line with the results of Llompart (2021b), who found that vocabulary size but not perception ability was a significant predictor of lexical encoding ability for advanced German-speaking learners of English, who had more accurate perception abilities, while perception ability but not vocabulary size was significant for intermediate learners, who had less accurate perception abilities. Furthermore, vocabulary size can be thought of as a proxy for proficiency and has been used as such in previous research (e.g., Darcy et al., 2016). It is probable that those learners with a higher proficiency level have had more L2 input, leading to more detailed and delineated representations because their exemplars are based on more examples. However, more input on its own would likely not be sufficient unless perception, or perhaps more accurately attentional cue weighing, is at a stage where exemplars can be fine-grained in terms of L2-relevant phonetic details. Otherwise, their exemplars are likely to reflect heavy influence from the L1 phonology (Maye, 2007). Perhaps additional analyses divided by proficiency level would reveal more about the effects of perception ability versus vocabulary size.

PSTM was also a significant factor in the current study, but for only the /tap-trill/ contrast when individual contrasts were examined, for which it explained slightly more variance than vocabulary size. Perhaps PSTM is important solely for the /tap-trill/ contrast because this is the only contrast in the current study in which the L2 sounds would overwhelmingly be assimilated to the same L1 sound, in this case English /ɹ/ (see Rose, 2012). Therefore, it may be that differences in phonological short-term memory come into play when sounds are differentiated along a dimension not used phonologically in the L1, making it more important to be able to hold finely detailed representations in the phonological loop long enough so that these L2-relevant details can be transferred to long-term representations. Because those with lower PSTM cannot hold phonetic details in memory for very long, when it comes time to convert the L2 sounds stored in the phonological loop into long-term representations, the memory traces may have degraded into less specific representations, such that these low-PSTM learners no longer retain a difference between the Spanish rhotics. Further research on the importance of PSTM for different types of contrasts could shed light on this question.

None of the regression analyses found a significant effect of inhibitory control or attention control. One possibility is that rather than directly impacting L2 representations, the effect goes in the opposite direction, and these cognitive abilities are instead enhanced by learning an L2. A wealth of research on bilingualism has generally found that bilingual individuals have stronger cognitive abilities than monolinguals, including attention control and inhibitory control (e.g., Bialystok et al., 2005; Adesope et al., 2010; Long et al., 2020). For example, Long and colleagues found that the Gaelic level of L2 learners predicted their attention switching ability, and improvement in L2 Gaelic skills corresponded to gains in attention switching (Long et al., 2020). Another possible explanation is that there was a problem with the specific tasks used in the current study or the way they were scored, since some participants displayed unexpected reaction time tendencies across conditions in both tasks. In fact, Hedge et al. (2018) argue that these kinds of widely-used cognitive tasks do not produce reliable individual differences in general. They state that tasks such as the flanker task became popular because of their reliable and easily replicable results at the group level, but this translates into low between-subject variability that is not reliably replicated across sessions. They found that none of the cognitive tasks they examined, including the flanker task, had reliability metrics at 0.8 or above, which is the accepted standard for clinical uses. Thus, more work may be needed in order to create more reliable tasks or more reliable ways of calculating scores for existing tasks in order to conduct valid individual differences research.

Overall, this study shows that L2 lexical encoding is affected by factors beyond perception, specifically L2 vocabulary size and phonological short-term memory. This corroborates previous research showing that learners’ phonolexical representations are fuzzy, above and beyond their ability to perceive the sounds within those words correctly. Additionally, this study reveals that the impact of individual differences depends on the contrast under examination, although acquiring a large vocabulary in the L2 appears to be the most important factor in mediating fuzzy lexical representations. Additional research is needed to determine if these results hold across other contrasts and language pairings, and to ascertain what other factors may be at play in the L2 lexical encoding of these contrasts, such as frequency, phonological neighborhood density, and phonetic variability in the input (see Llompart (2021a), under this Research Topic).

## Data Availability Statement

The datasets and analyses presented in this study can be found in the Open Science Framework repository at osf.io/w9mnr.

## Ethics Statement

The studies involving human participants were reviewed and approved by the Human Research Protection Program (HRPP) at Indiana University. Written informed consent for participation was not required for this study in accordance with the national legislation and the institutional requirements.

## Author Contributions

As this article describes part of DD’s dissertation research, she was the main contributor for all parts of this study and she alone performed the data collection. ID was the advisor for this work and aided in conceptualization and design of the experiment as well as the analyses and interpretation of the results. DD wrote the first draft of the manuscript, which ID revised and expanded. Both authors approved the final submitted version.

## Conflict of Interest

The authors declare that the research was conducted in the absence of any commercial or financial relationships that could be construed as a potential conflict of interest.

## Footnotes

1 A small number of native Spanish speakers were also tested in order to ensure that the tasks were working as expected. See Daidone (2020) for details.

2 For an in-depth review of the major L2 phonological models and their connection to lexical encoding, see Daidone (2020).
