# Supplementary material for: Vocabulary Size Is a Key Factor in Predicting Second Language Lexical Encoding Accuracy
Source: Front Psychol. 2021 Jul 22;12:688356. doi: 10.3389/fpsyg.2021.688356 (PMC8339215; doi:10.3389/fpsyg.2021.688356)
Supplement: Supplementary file 1 [file Table_1.DOCX]

# Appendix A: Stimuli for the lexical decision task

Test stimuli for lexical decision task, List 1

| **/tap-*trill/** | **/trill-*tap/** | **/tap-*d/** | **/d-*tap/** | **/trill-*d/** | **/d-*trill/** |
| --- | --- | --- | --- | --- | --- |
| **Word** | **Word** | **Word** | **Word** | **Word** | **Word** |
| dinero | correcto | general | miedo | ocurre | estado |
| sería | aburrido | corazón | adelante | corre | partido |
| primero | arroz | cultura | edificio | cierra | medio |
| durante | arriba | daría | comida | guerra | nadie |
| quiero | tierra | historia | sonido | corrige | todavía |
| **Nonword** | **Nonword** | **Nonword** | **Nonword** | **Nonword** | **Nonword** |
| señorra | eror | maneda | mericina | desadollo | esturrio |
| gustarría | horible | dedecha | abogaro | nadativa | larro |
| mirro | interumpe | clado | sábaro | codiente | pasarro |
| diferrente | aranca | fueda | mérico | adegla | demasiarro |
| parrece | párafo | númedo | vestiro | tedible | ayurra |

Control stimuli for lexical decision task, List 1

| **/f-*p/** | **/p-*f/** |
| --- | --- |
| **Word** | **Word** |
| difícil | grupo |
| jefe | guapo |
| oficina | deporte |
| uniforme | capital |
| teléfono | lápiz |
| **Nonword** | **Nonword** |
| epecto | pafel |
| gapas | refente |
| reporma | afenas |
| apuera | cafaz |
| signipica | zafato |

Test stimuli for lexical decision task, List 2

| **/tap-*trill/** | **/trill-*tap/** | **/tap-*d/** | **/d-*tap/** | **/trill-*d/** | **/d-*trill/** |
| --- | --- | --- | --- | --- | --- |
| **Word** | **Word** | **Word** | **Word** | **Word** | **Word** |
| señora | error | manera | medicina | desarrollo | estudio |
| gustaría | horrible | derecha | abogado | narrativa | lado |
| miro | interrumpe | claro | sábado | corriente | pasado |
| diferente | arranca | fuera | médico | arregla | demasiado |
| parece | párrafo | número | vestido | terrible | ayuda |
| **Nonword** | **Nonword** | **Nonword** | **Nonword** | **Nonword** | **Nonword** |
| dinerro | corecto | genedal | miero | ocude | estarro |
| serría | aburido | codazón | arelante | code | partirro |
| primerro | aroz | cultuda | erificio | cieda | merrio |
| durrante | ariba | dadía | comira | gueda | narrie |
| quierro | tiera | histodia | soniro | codige | torravía |

Control stimuli for lexical decision task, List 2

| **/f-*p/** | **/p-*f/** |
| --- | --- |
| **Word** | **Word** |
| efecto | papel |
| gafas | repente |
| reforma | apenas |
| afuera | capaz |
| significa | zapato |
| **Nonword** | **Nonword** |
| dipícil | grufo |
| jepe | guafo |
| opicina | deforte |
| uniporme | cafital |
| telépono | láfiz |

Practice and filler stimuli for lexical decision task, both List 1 and List 2

| **Practice** | **Practice** | **Filler** | | | **Filler** | | |
| --- | --- | --- | --- | --- | --- | --- | --- |
| **Word** | **Nonwords** | **Word** | | | **Nonword** | | |
| cama | hermoto | cabeza | vuelo | noche | bigue | leto | niecha |
| lago | querto | rata | avión | para | blario | mabio | fendo |
| verde | jeso | actor | banco | pie | bundad | jestu | flío |
| madera | pieno | listo | todo | llama | cheijo | chempo | pengo |
| postre | bepa | mata | voy | antes | chelpo | mesque | ganafe |
|  |  | batalla | escuela | seis | diano | tefpo | gaque |
|  |  | plato | clase | come | faufe | nano | gaufo |
|  |  | gato | mañana | siente | fella | nante | guepo |

# Appendix B: Stimuli for the oddity task

Test and control stimuli for oddity task

| Condition | Contrast | Stimuli | | | |
| --- | --- | --- | --- | --- | --- |
|  |  | A | | B | |
|  |  | Orthography | IPA | Orthography | IPA |
| Test | /ɾ-r/ | quira | /ˈki.ɾa/ | quirra | /ˈki.ra/ |
|  |  | nera | /ˈne.ɾa/ | nerra | /ˈne.ra/ |
|  |  | cuare | /ˈku̯a.ɾe/ | cuarre | /ˈku̯a.re/ |
| Test | /ɾ-d/ | fare | /ˈfa.ɾe/ | fade | /ˈfa.de/ |
|  |  | mare | /ˈma.ɾe/ | made | /ˈma.de/ |
|  |  | liero | /ˈli̯e.ɾo/ | liedo | /ˈli̯e.do/ |
| Test | /r-d/ | cherra | /ˈtʃe.ra/ | cheda | /ˈtʃe.da/ |
|  |  | terro | /ˈte.ro/ | tedo | /ˈte.do/ |
|  |  | morre | /ˈmo.re/ | mode | /ˈmo.de/ |
| Control | /f-p/ | lefo | /ˈle.fo/ | lepo | /ˈle.po/ |
|  |  | mafe | /ˈma.fe/ | mape | /ˈma.pe/ |
|  |  | quefe | /ˈke.fe/ | quepe | /ˈke.pe/ |

Filler stimuli for oddity task

| Condition | Contrast | Stimuli | | | |
| --- | --- | --- | --- | --- | --- |
|  |  | A | | B | |
| Filler |  | Orthography | IPA | Orthography | IPA |
|  | /ʝ-l/ | nella | /ˈne.ʝa/ | nela | /ˈne.la/ |
|  | /s-l/ | lespo | /ˈles.po/ | lelpo | /ˈlel.po/ |
|  | /a-ai̯/ | came | /ˈka.me/ | caime | /ˈkai̯.me/ |
|  | /d-t/ | chade | /ˈtʃa.de/ | chate | /ˈtʃa.te/ |
|  | /a-e/ | nalco | /ˈnal.ko/ | nelco | /ˈnel.ko/ |
|  |  | fega | /ˈfe.ga/ | fegue | /ˈfe.ge/ |

# Appendix C: Stimuli for the phonological short-term memory task

Stimuli for practice trials in the phonological short-term memory task

| Type of trial | Stimulus 1 | Stimulus 2 | Stimulus 3 | Stimulus 4 |
| --- | --- | --- | --- | --- |
| same | meht | pyehk | syash | vohm |
| same | pyesh | vyat | dohs | mehr |
| different | doht | **syehm** | **lyas** | poht |
| different | sohr | **pish** | **vahm** | lohr |

*Note.* Stimuli were presented in the same order for both iterations in “same” trials. Stimuli that are bolded are those that were switched for the second iteration in “different” trials.

Stimuli for sequences of length 4 in the phonological short-term memory task

| Type of trial | Stimulus 1 | Stimulus 2 | Stimulus 3 | Stimulus 4 |
| --- | --- | --- | --- | --- |
| same | mar | pohl | siehr | lyat |
| same | lahl | mohm | dyak | lyehch |
| same | mohl | vyash | vis | sahl |
| same | vyehsh | dohr | lil | mam |
| different | dim | **pyal** | **syehk** | mahch |
| different | mis | **dahk** | **lyeht** | sir |
| different | dahs | **myal** | **tohm** | pahk |
| different | mich | **lyehk** | **pahsh** | vit |

*Note.* Stimuli were presented in the same order for both iterations in “same” trials. Stimuli that are bolded are those that were switched for the second iteration in “different” trials.

Stimuli for sequences of length 5 in the phonological short-term memory task

| Type of trial | Stimulus 1 | Stimulus 2 | Stimulus 3 | Stimulus 4 | Stimulus 5 |
| --- | --- | --- | --- | --- | --- |
| same | myach | pil | vohr | pahs | lyehsh |
| same | dyam | sohm | pir | pohs | mohsh |
| same | sahch | syak | lyam | vyehm | lohs |
| same | dohch | vohl | vyar | myas | sish |
| different | pohch | **syal** | **mik** | syehm | pyehr |
| different | vohch | vahl | **pyam** | **dir** | sohk |
| different | lich | **pim** | **lyehl** | vahr | pyesh |
| different | sohch | vyehk | **vahs** | **myehl** | dyash |

*Note.* Stimuli were presented in the same order for both iterations in “same” trials. Stimuli that are bolded are those that were switched for the second iteration in “different” trials.

Stimuli for sequences of length 6 in the phonological short-term memory task

| Type of trial | Stimulus 1 | Stimulus 2 | Stimulus 3 | Stimulus 4 | Stimulus 5 | Stimulus 6 |
| --- | --- | --- | --- | --- | --- | --- |
| same | dyach | mahk | sim | lahr | syehs | myash |
| same | sich | lehk | lyal | pahm | sohs | pyash |
| same | syehch | dohk | sil | myehm | lyar | lahsh |
| same | lyach | pyak | dahl | lim | vyehr | syas |
| different | vehl | lahk | **lohm** | **pyach** | sis | dyehr |
| different | dil | **vohk** | **pyehm** | mohch | pahr | vyas |
| different | vyehch | mim | sik | **dyehl** | **mehsh** | lehs |
| different | vil | sahm | **pyar** | **myak** | dis | mehch |

*Note.* Stimuli were presented in the same order for both iterations in “same” trials. Stimuli that are bolded are those that were switched for the second iteration in “different” trials.

Stimuli for sequences of length 7 in the phonological short-term memory task

| Type of trial | Stimulus 1 | Stimulus 2 | Stimulus 3 | Stimulus 4 | Stimulus 5 | Stimulus 6 | Stimulus 7 |
| --- | --- | --- | --- | --- | --- | --- | --- |
| same | pohr | dyehch | vyam | lik | sahs | lyash | dohl |
| same | vyach | lehm | vir | syehl | pich | dyas | sahsh |
| same | lyehr | pyehch | mash | vik | mil | mam | lahs |
| same | vich | lohk | pyehl | lahm | dahr | vohs | dyehsh |
| different | pyehk | **lahch** | **sohl** | sahr | dohm | pis | vish |
| different | pyehs | dahch | pik | **vyal** | **syehsh** | lir | dyehm |
| different | vim | mohr | dyehs | myam | **vahk** | **dich** | lohl |
| different | lil | pohk | **dahm** | **mir** | pahch | sohsh | lis |

*Note.* Stimuli were presented in the same order for both iterations in “same” trials. Stimuli that are bolded are those that were switched for the second iteration in “different” trials.

# Appendix D: Stimuli for the retrieval-induced inhibition task

Table 45. Stimuli for the retrieval-induced inhibition task

| Words for Memorization, Practice, & Test Phases | Distractor Words for the Test Phase |
| --- | --- |
| ANIMALS - horse  ANIMALS - elephant  ANIMALS - tiger  ANIMALS - duck  ANIMALS - cow  ANIMALS - snake | donkey  giraffe  deer  lion  rabbit  zebra |
| OCCUPATIONS - nurse  OCCUPATIONS - teacher  OCCUPATIONS - engineer  OCCUPATIONS - dentist  OCCUPATIONS - carpenter  OCCUPATIONS - firefighter | mechanic  policeman  secretary  farmer  cook  lawyer |
| FRUITS - grape  FRUITS - apple  FRUITS - orange  FRUITS - pear  FRUITS - cherry  FRUITS - raspberry | blueberry  coconut  plum  mango  papaya  fig |
